# Apigenin Inhibits Histamine-Induced Cervical Cancer Tumor Growth by Regulating Estrogen Receptor Expression

**DOI:** 10.3390/molecules25081960

**Published:** 2020-04-23

**Authors:** Erkang Zhang, Yani Zhang, Zhuoyan Fan, Lei Cheng, Shiwen Han, Huilian Che

**Affiliations:** Beijing Advanced Innovation Center for Food Nutrition and Human Health, College of Food Science and Nutritional Engineering, China Agricultural University, Beijing 100083, China

**Keywords:** apigenin, cervical cancer, histamine, estrogen receptor, PI3K/Akt/mTOR pathway

## Abstract

Apigenin is a natural flavone with anti-inflammatory and antioxidant properties and antitumor abilities against several types of cancers. Previous studies have found that the antitumor effects of apigenin may be due to its similar chemical structure to 17β-estradiol (E2), a main kind of estrogen in women. However, the precise mechanism underlying the antitumor effects of apigenin in cervical cancer remains unknown. On the other hand, there is increasing evidence that describes a histamine role in cancer cell proliferation. In this study, we examined whether apigenin can attenuate the effects of histamine on tumors by regulating the expression level of estrogen receptors (ERs) to inhibit cervical cancer growth. Our in vitro data indicates that apigenin inhibited cell proliferation in a dose-dependent manner in human cervical cancer cells (HeLa), while histamine shows the opposite effects. After that, the xenograft model was established to explore the antitumor effects of apigenin in vivo, the results show that apigenin inhibited cervical tumor growth by reversing the abnormal ER signal in tumor tissue which was caused by histamine. We also demonstrate that apigenin inhibited cell proliferation via suppressing the PI3K/Akt/mTOR signaling pathway. Collectively, our results suggest that apigenin may inhibit tumor growth through the ER-mediated PI3K/Akt/mTOR pathway and that it can also attenuate the effects of histamine on tumors.

## 1. Introduction

Cervical cancer, as a gynecological tumor, remains one of the most common causes of cancer-related deaths worldwide, which consists of 528,000 new cases and 266,000 deaths worldwide each year [1,2]. Most of the cervical cancer cases result from infection with human papillomavirus, and the primary treatment options for these patients include surgery followed by radiotherapy [3]. Nevertheless, poor prognosis is attributed to their toxic side effects and the increased incidence of other risk factors, such as vascular space invasion and lymph node metastases [4]. On the other hand, in those underdeveloped countries which grossly lack sufficient treatment, cervical cancer causes more than one quarter of a million deaths per year [5]. Fortunately, various bioactive natural compounds have already been well studied and shown to be useful in prevention and therapy of cancer by regulating numerous signaling pathways. According to a previous published review, drugs targeting cancer approved by the United States Food and Drug Administration during the last three decades were either natural products per se or based thereon [6]. Therefore, a natural product with better curative effects and lower side effects should be found as a potential therapy method for cervical cancer.

Apigenin (4′,5,7-trihydroxyflavone) is a naturally occurring plant flavone, widely present in fruits and vegetables, including onions, oranges, tea and some seasonings [7]. Recently, apigenin has been recognized as a bioactive flavonoid shown to possess anti-inflammatory, antioxidant and anticancer properties [8,9]. Interestingly, apigenin is also one of the most abundantly distributed botanical phytoestrogens, having a similar chemical structure to 17β-estradiol (E2) [10]. This characteristic of the chemical structure suggests that apigenin may affect the development of estrogen-related cancer, such as cervical cancer, by regulating the endocrine system. Many previous studies results support this hypothesis, researchers have found that phytoestrogens can inhibit the growth and proliferation of cancer cells [11,12]. However, the precise mechanism of the anti-tumor effects of apigenin remains poorly understood, especially when it comes to cervical tumors.

Tumor development and progression relies on cell proliferation, and high levels of histamine and its synthetizing enzyme are found in growing tumor tissues [13]. Specifically, histamine induces cell proliferation through four histamine receptors (HRH1, HRH2, HRH3 and HRH4) which differ in tissue expression profiles and functions [14,15]. In prostate cancer, another cancer which is related to the endocrine system, histamine receptors were found to be able to change the expression level of androgen receptors in cancer tissues [16]. Accordingly, when it comes to cervical cancer we should figure out how histamine influences the female endocrine system and leads to a progression of cervical cancer. Furthermore, too little work has been devoted to exploring the effects of apigenin on cervical cancer, especially as a kind of phytoestrogen.

In this paper, we establish in vivo and in vitro studies to investigate the mechanism of the effects of histamine and apigenin on cervical cancer. Our results indicate that histamine promoted tumor growth, while apigenin showed the opposite effects. Additionally, we foun that the histamine-induced tumor growth resulted from the expression level change of ERα and ERβ, which is an abnormal estrogen receptor (ER) signal, but apigenin could reverse this effect. We also demonstrated that both apigenin and histamine could influence the autophagy and apoptosis of HeLa cells via regulating the PI3K/Akt/mTOR signaling pathway.

## 2. Results

### 2.1. Histamine Treatment Stimulate Cervical Cancer Tumor Growth In Vivo and In Vitro

**Figure 1:**
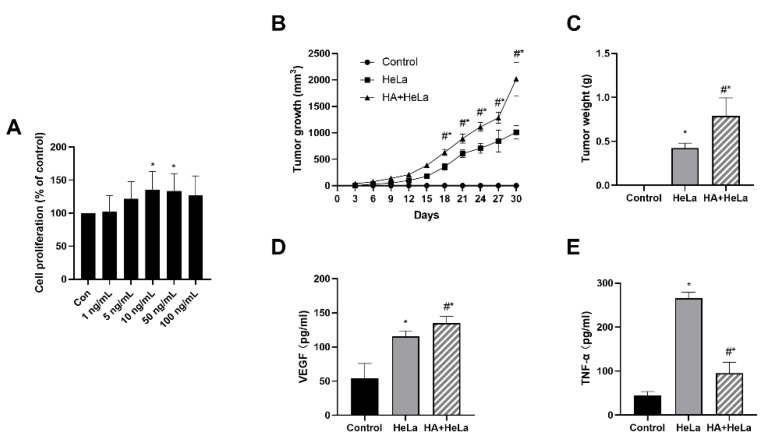
Histamine treatment induced cervical cancer growth in vivo and in vitro. HeLa cells were injected subcutaneously into the lower right side of female BALB/c nude mice. After 6 days, mice were randomly divided into three groups: control, HeLa (no histamine), HA + HeLa (1 mg/kg). Animals were intraperitoneally injected with histamine or PBS (same volume as histamine treatment) every 3 days. (**A**) HeLa cell proliferation after treatment with different concentrations of histamine (1, 5, 10, 50, 100 ng/mL). HeLa cells were incubated with histamine for 48 h. (**B**) Average tumor volume was measured every 3 days. (**C**) Tumor weights were measured at the end of treatment. (**D**) ELISA analysis for serum VEGF levels. (**E**) ELISA analysis for serum TNF-α levels. The serum used in (**D**,**E**) was obtained by the orbital sinus of mice. Histamine promoted cervical cancer growth according to the tumor growth and tumor weight. Data are presented as the mean ± SEM of at least three independent experiments. * *p* < 0.05 versus the control group, # *p* < 0.05 versus the HeLa group.

To investigate the effects of histamine on tumor growth in vitro, we treated HeLa cells with different concentrations of histamine (1, 5, 10, 50, 100 ng/mL). After incubation with histamine for 48 h, we found that the proliferation of HeLa cells was stimulated by histamine in a dose-dependent manner between the concentration of 1 ng/mL and 10 ng/mL (Figure 1A). Histamine concentrations at 10 and 50 ng/mL significantly stimulated the proliferation of HeLa cells. Interestingly, the cell proliferation was slightly lower in 100 ng/mL compared to 50 ng/mL, this probably can be explained by the cytotoxicity of histamine at a high concentration. To further examine whether histamine could induce tumor growth in vivo, we established the xenograft model in female BALB/c nude mice and the mice were intraperitoneally injected with 1 mg/kg of histamine every three days. As shown in Figure 1B,C, histamine administration induced tumor growth compared with the HeLa group. The tumor volume and tumor weight were remarkably higher in the group treated with histamine. Additionally, serum VEGF and TNF-α levels were measured using ELISA assays. Serum VEGF levels were significantly higher in the HA + HeLa group, which indicates that the injection of histamine may have induced the vascular endothelial growth in nude mice (Figure 1D). On the contrary, TNF-α levels were found to be dramatically decreased in the histamine treatment group (Figure 1E). Taken together, our findings demonstrate that histamine promoted cervical cancer tumor growth in vivo and in vitro.

### 2.2. Histamine Induced Cervical Tumor Growth by Altering the Expression of Estrogen Receptor

**Figure 2:**
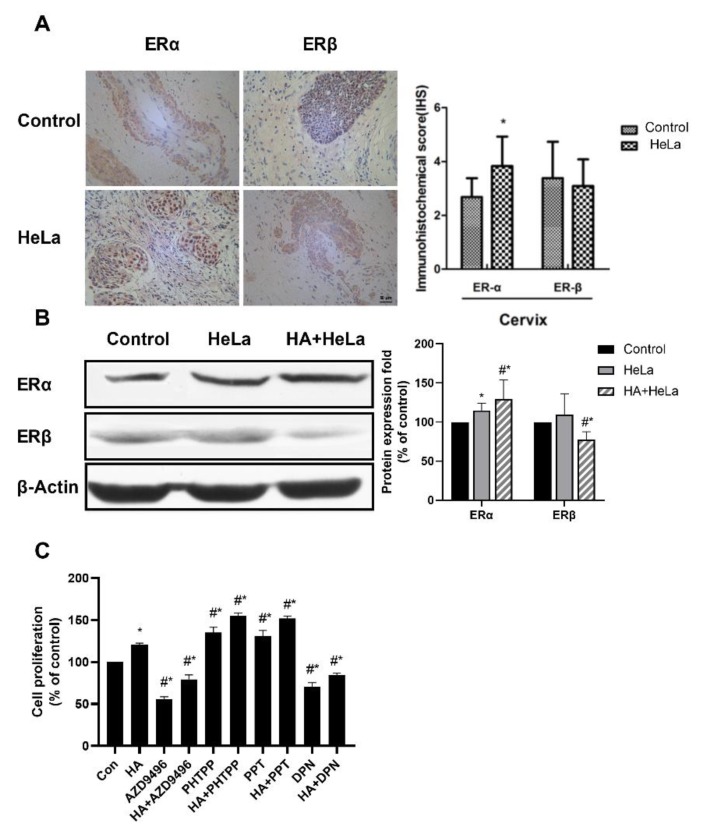
Histamine induced tumor growth by altering the expression level of the estrogen receptors (ERs). The expression of ERα was promoted after treatment with histamine, while ERβ showed the opposite results. (**A**) Immunohistochemistry detection of ER expression in the cervical tissue of the control group and HeLa group. (**B**) Western blot analysis of the expression levels of ERα and ERβ from respective tumor tissues. The results are expressed as a percentage of control, which is set at 100%. The antibodies used in this experiment were ERα (ab32063) and ERβ (ab288) (**C**) Effects of four kinds of ER modulator (AZD9496, PHTPP, PPT and DPN) and HA on HeLa cell proliferation, HeLa cells were incubated with these reagents at the concentration of 1 μM and the fortification concentration of HA was 50 ng/mL. All cells were incubated with histamine and ER modulators for 48 h before the proliferation percentage was measured. Cas registry number for ER modulators: AZD9496 (CAS No.: 1639042-08-2), PHTPP (CAS No.: 805239-56-9), PPT (CAS No.: 263717-53-9) and DPN (CAS No.: 1428-67-7). Data are presented as the mean ± SEM of at least three independent experiments. * *p* < 0.05 versus the control group. # *p* < 0.05 versus the HeLa or HA group

Many gynecologic oncologies, such as breast cancer, are considered ER-positive, indicating the correlation between ER and tumor growth. Therefore, the ERα and ERβ expression levels in normal and cervical cancer tissues were measured by immunohistochemistry. As shown in Figure 2A, when compared to normal tissue the expression of ERα in HeLa group was significantly higher, while the expression of ERβ remained roughly unchanged. Then, Western blot analysis was established to investigate the ER expression in xenograft nude mice. Results showed that ERα expression was upregulated in tumor tissue while the expression of ERβ was decreased, and that histamine treatment enhanced this effect (Figure 2B). According to this, histamine induced an abnormal ER signal which probably resulted in the change in tumor growth. To further ascertain whether the ER expression change caused by histamine promoted cervical tumor growth, HeLa cells were treated with AZD9496 (an ERα inhibitor), PHPPT (an ERβ inhibitor), PPT (an ERα agonist) or DPN (an ERβ agonist) for 48 h. After that, the proliferation of HeLa cells was measured by Cell Counting Kit-8 (CCK-8) assays. Our results demonstrate that PPT and PHPPT showed a significant promotion effect on cell proliferation, while AZD9496 and DPN showed an opposite effect (Figure 2C). So, it can be clearly deduced that the expression change of ER caused by histamine could further influence cell proliferation. Taken together, these results suggest that histamine induced the cervical tumor growth by altering the expression of ERα and ERβ. 

### 2.3. The PI3K/Akt/mTOR Pathway Is Activated by Histamine

**Figure 3:**
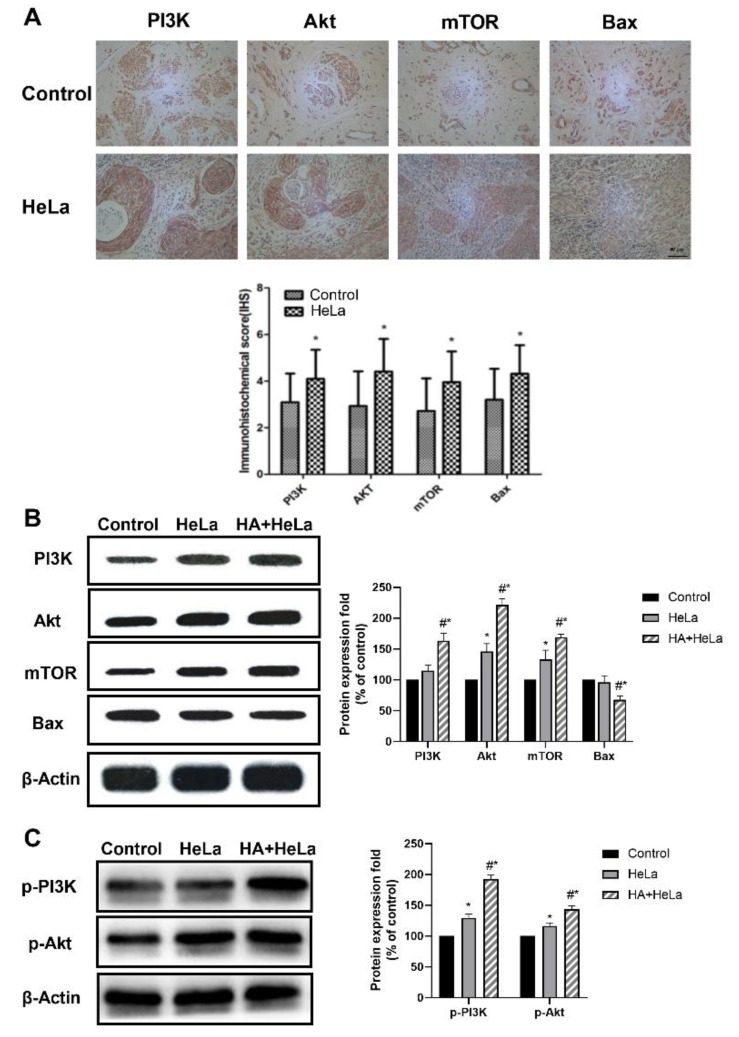
Histamine induced apoptosis via inhibition of the PI3K/Akt/mTOR signaling pathway in HeLa cells. The PI3K/Akt/mTOR signaling pathway was activated by the treatment of histamine. (**A**) Immunohistochemistry detection of PI3K, Akt, mTOR and Bax expression level in the cervical tissue of the control group and HeLa group. (**B**) Western blot analysis of PI3K, Akt, mTOR and Bax expression level from respective tumor tissues. The antibodies used in this experiment were PI3K (ABP52199), Akt (ABM40276), mTOR (ABP51866) and Bax (ABP55948). (**C**) Western blot analysis of p-PI3K and p-AKT expression level from respective tumor tissues. The antibodies used in this experiment were p-PI3K(ab182651) and p-Akt(ab81283). The locus of p-PI3K and p-Akt was Y607 and S473 respectively. The results are expressed as a percentage of control, which is set at 100%. Data are presented as the mean ± SEM of at least three independent experiments. * *p* < 0.05 versus the control group, # *p* < 0.05 versus the HeLa group.

The PI3K/Akt/mTOR signaling pathway plays an important role in regulating the cell cycle, proliferation and apoptosis. Significant increases in the expression of PI3K, Akt and mTOR, determined by immunohistochemistry analysis, were observed in the cervical tumor tissue (Figure 3A). Western blot experiments were established to further investigate the effect of histamine on the PI3K/Akt/mTOR pathway. The results revealed that protein levels in tumor tissue were higher after treatment with histamine (Figure 3B), indicating that the treatment with histamine suppressed cell apoptosis via the PI3K/Akt/mTOR pathway, which leads to the positive influence of histamine on cervical cancer development. On the other hand, the expression level of Bax, a crucial pro-apoptotic protein, was found to be decreased in HA + HeLa group (Figure 3B). Additionally, the upstream proteins can activate downstream proteins by phosphorylation, so we decided to investigate the expression level of p-PI3K and p-Akt. In Figure 3C, we found that the phosphorylation of PI3K and Akt were also significantly increased after the administration of histamine. Collectively, these data suggest that histamine promoted the cervical tumor growth through the activation of the PI3K/Akt/mTOR pathway.

### 2.4. Inhibition Effects of Apigenin on HeLa Cell Proliferation

**Figure 4:**
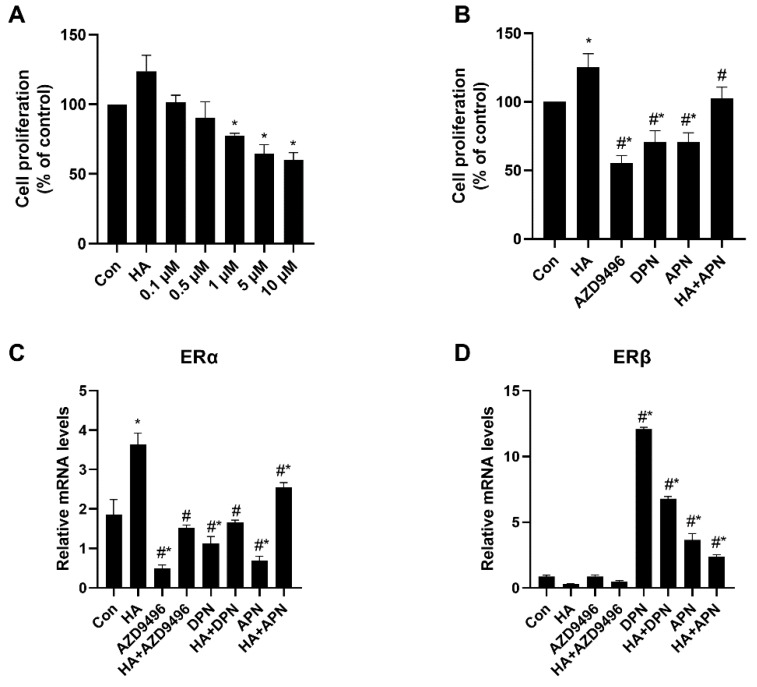
Inhibition effects of apigenin on HeLa cell proliferation. (**A**) HeLa cell proliferation after treated with different concentration of apigenin (0.1, 0.5, 1, 5, 10 μM). Cell proliferation was measured after incubation with apigenin for 48 h. (**B**) Effects of HA, AZD9496, DPN and apigenin treatment on HeLa cell proliferation. The concentration of AZD9496 and DPN was 1 μM. (**C**) The mRNA expression levels of ERα. (**D**) The mRNA expression levels of ERβ. The results are expressed as a percentage of control, which is set at 100%. The concentration of apigenin used in (**B**–**D**) was 5 μM. The detailed primer sequence used in this part is shown in the materials and methods section. Data are presented as the mean ± SEM of at least three independent experiments. * *p* < 0.05 versus the control group, # *p* < 0.05 versus the HA group.

HeLa cells were incubated with different concentrations of apigenin (0.1, 0.5, 1, 5, 10 μM) for 48 h, and cell proliferation was measured by CCK-8 assays. As shown in Figure 4A, apigenin inhibited HeLa cell proliferation in a dose-dependent manner. As is expected, both AZD9496 and DPN significantly inhibited the HeLa cell proliferation and their inhibition effects were similar to apigenin (Figure 4B). We hypothesized that apigenin suppressed HeLa cell proliferation by altering ER signal, so the mRNA release level of genes coding ERα and ERβ in cells was assessed. Interestingly, the expression of ERα and ERβ genes were dramatically changed by apigenin (Figure 4C,D), which also probably explains the inhibition effects of AZD9496 and DPN, two kinds of estrogen receptor modulator similar to apigenin. These data indicate that apigenin may have inhibited the HeLa cell proliferation in vitro, suggesting the potential regulating mechanism between apigenin and ER.

### 2.5. Apigenin Inhibited Cervical Tumor Growth In Vivo by Attenuating the Abnormal ER Signaling Caused by Histamine

**Figure 5:**
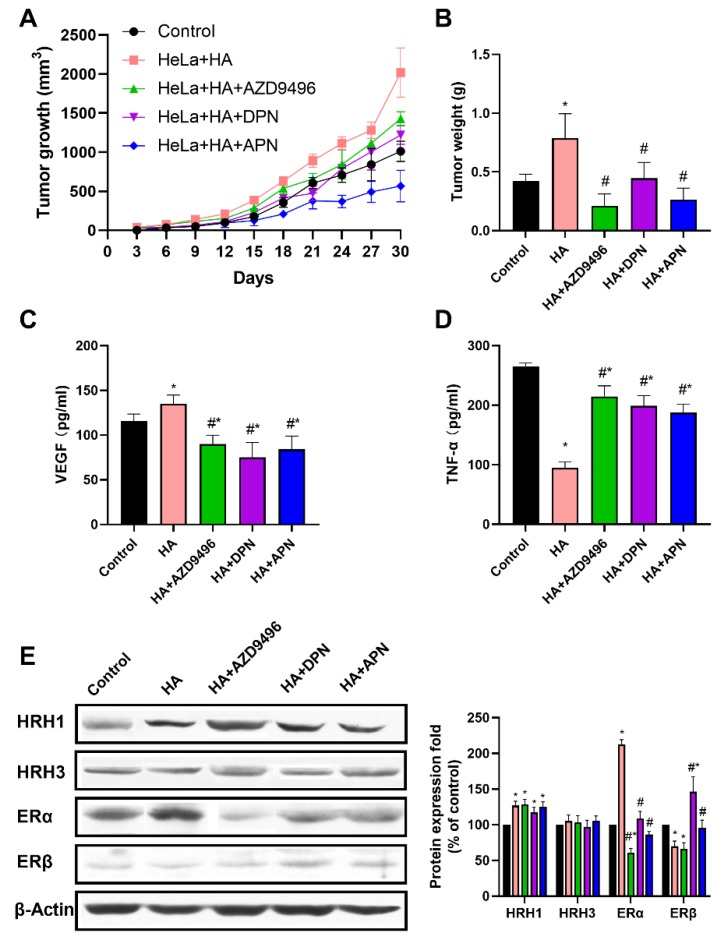
Apigenin inhibited cervical tumor growth in vivo by attenuating the abnormal ER signaling caused by histamine. HeLa cells were injected subcutaneously into the lower right side of female BALB/c nude mice. After 6 days, mice were divided into five groups; control, HeLa + HA (1 mg/kg), HA + AZD9496 (1 mg/kg + 2 mg/kg), HA + DPN (1 mg/kg + 2 mg/kg) and HA + APN (1 mg/kg + 100 mg/kg). Animals were intraperitoneally injected with these reagents every 3 days. Effects of apigenin on cervical tumor growth was determined by the tumor volume and tumor weight. (**A**) Average tumor volume was measured every 3 days. (**B**) Tumor weights were measured at the end of treatment. (**C**) ELISA analysis for serum VEGF levels. (**D**) ELISA analysis for serum TNF-α levels. (**E**) Western blot analysis on the expression level of ERα, ERβ, HRH1 and HRH3 from respective tumor tissues. The antibodies used in this experiment were ERα (ab32063), ERβ (ab288), HRH1 (ABP51517) and HRH3 (ABP53626). The results are expressed as a percentage of control, which is set at 100%. The serum used in (**C**,**D**) was obtained from the orbital sinus of mice. Data are presented as the mean ± SEM of at least three independent experiments. * *p* < 0.05 versus the control group, # *p* < 0.05 versus the HA group.

Next, we determined whether apigenin can inhibit cervical tumor growth in the xenograft model in female BALB/c nude mice. Compared with control group and histamine treatment group, tumor volume and tumor weight were found to be obviously lower in groups injected with apigenin, AZD9496 and DPN (Figure 5A,B). We also detected the serum VEGF and TNF-α levels using ELISA assays. As shown in Figure 5C,D, the release of VEGF was inhibited in the apigenin, AZD9496 and DPN treatment groups, but the serum TNF-α level in these groups was significantly increased compared with the group treated with histamine alone. To clarify whether apigenin could reverse the abnormal ER expression level caused by histamine, we performed Western blot analysis to detect the expression level of ER in tumor tissue. As shown in Figure 5E, apigenin administration significantly attenuated the abnormal expression level of ERα and ERβ induced by histamine. Compared with mice treated with histamine alone, the ERα expression of mice in AZD9496, DPN and APN group was downregulated, while expression of ERβ was upregulated. In addition, the expression of two kinds of main histamine receptor (HRH1 and HRH3) in cervical tumors was measured to ascertain that apigenin did not alter the ER signal by protecting HeLa cells from receiving histamine signal. As is expected, Western blot results show that apigenin treatment did not change the expression level of HRH1 and HRH3 (Figure 5E). Overall, these results demonstrate that apigenin inhibited cervical tumor growth in vivo by attenuating the abnormal ER signaling caused by histamine.

### 2.6. PI3K/Akt/mTOR Pathway Was Inhibited by the ER Expression Level Change Caused by Apigenin Treatment

**Figure 6:**
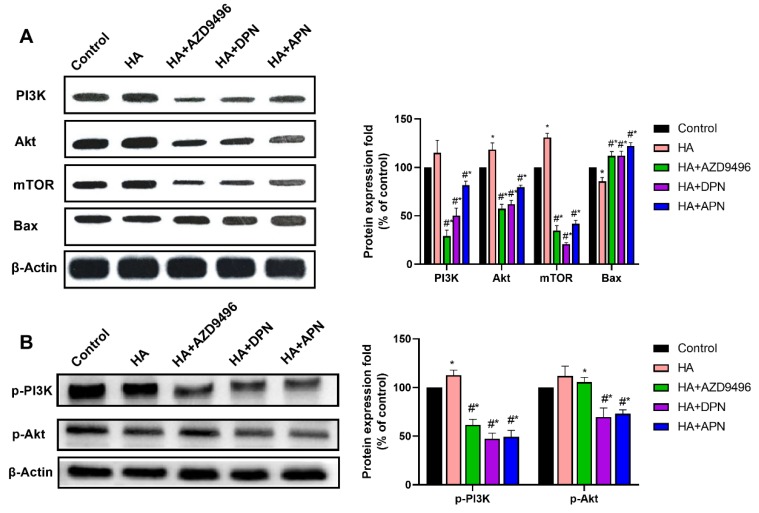
(**A**) Western blot analysis of PI3K, Akt, mTOR and Bax expression level from respective tumor tissues. The antibodies used in this experiment were PI3K (ABP52199), Akt (ABM40276), mTOR (ABP51866) and Bax (ABP55948). (**B**) Western blot analysis of p-PI3K and p-Akt expression levels from respective tumor tissues. The antibodies used in this experiment were p-PI3K(ab182651) and p-Akt(ab81283). The locus of p-PI3K and p-Akt was Y607 and S473 respectively. The results are expressed as a percentage of control, which is set at 100%. Data are presented as the mean ± SEM of at least three independent experiments. * *p* < 0.05 versus the control group, # *p* < 0.05 versus the HA group.

In this part, we investigated whether the expression change of ER caused by apigenin could inhibit the PI3K/Akt/mTOR signaling pathway, thereby suppressing tumor development. After treatment with apigenin, the activation of PI3K/Akt/mTOR signaling pathway caused by histamine was attenuated, and the expression of Bax was increased (Figure 6A). Further to this, AZD9496 and DPN, the ER modulators which influenced the function of ERα and ERβ respectively, also showed inhibition effects on the expression of PI3K, Akt, and mTOR. Apart from this, our results demonstrate that apigenin also significantly attenuated the activation effects of histamine on p-PI3K and p-Akt. These findings indicate that apigenin can suppress the PI3K/Akt/mTOR signaling pathway by altering expression of ER, thus inducing apoptosis and autophagy in HeLa cells.

### 2.7. Apigenin Modulated the Serum Estradiol Level in Mice

**Figure 7:**
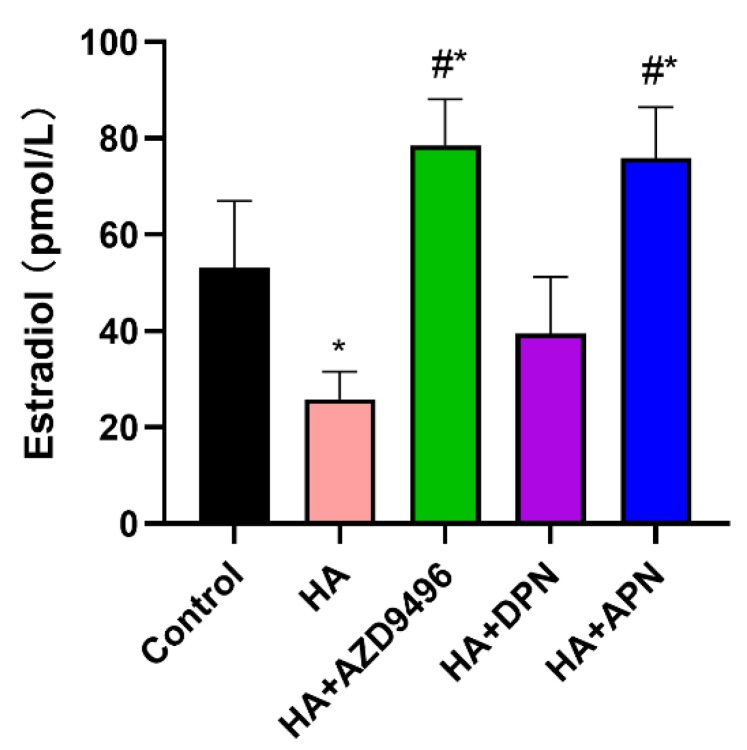
ELISA analysis for serum estradiol level. Fortification of APN and AZD9496 significantly increased serum 17β-estradiol (E2) levels. The serum used in this experiment was obtained from the orbital sinus of mice. Data are presented as the mean ± SEM of at least three independent experiments. * *p* < 0.05 versus the control group, # *p* < 0.05 versus the HA group.

Apigenin, which has a similar chemical structure to E2, was supposed to influence the endocrine system in nude mice. Therefore, we assessed serum estradiol level in nude mice. Histamine treatment significantly decreased the E2 concentration in serum while the fortification of AZD9496 and apigenin reversed this effect, which is probably due to the competitive binding of apigenin and estradiol to ERα (Figure 7).

## 3. Discussion

The development of cervical cancer, as a kind of hormone dependent cancer, was believed to be closely related to the body’s hormone and receptor levels [17,18]. Recent evidence has indicated that histamine can induce the proliferation of prostate cancer cells by influencing the expression level of androgen receptors [16]. It is also very well known that the immune system plays a crucial role with respect to the development as well as resolution of malignant lesions. A previous study showed that histamine secreted by mast cells triggers cell proliferation, embryonic development and tumor growth [19]. We found that histamine can induce HeLa cell proliferation by causing abnormal ER signal. Following the same train of thought, we hypothesized that apigenin attenuated the effects of histamine on tumors by regulating the expression level of estrogen receptors to inhibit cervical cancer growth. As is expected, our data support this hypothesis.

Regarding the contribution to tumor progression, pro-inflammatory factors, growth factors and immune factors are the best-characterized soluble microenvironmental factors [20]. According to several papers, histamine becomes an autocrine growth factor that regulates cell proliferation via HRH1 in experimental mammary carcinomas [21,22,23]. In another hormone-dependent cancer, prostate cancer, HRH3 was found to be overexpressed in prostate cancer tissue and associated with cell proliferation [16]. As previously mentioned, our data are consistent with their conclusion that histamine can induce HeLa cell proliferation in a dose-dependent manner. Moreover, in the in vivo study, we determined that the promotion effects of histamine on cervical cancer is based on the ER signal. To the best of our knowledge, this is the first study that reports that histamine induces HeLa cell proliferation by influencing ER expression.

Previous studies have shown that apigenin can inhibit cell proliferation and induce apoptosis: Yang et al. reported that apigenin suppressed proliferation of HeLa cells in a dose-dependent manner and caused apoptosis through PI3K/Akt/mTOR signaling pathway [24]. Chiang et al. reported that apigenin exhibited an anti-proliferation and induced apoptosis in human Hep G2 cells [25]. Likewise, we have found that the administration of apigenin caused proliferation inhibition in HeLa cells. We then investigated whether apigenin could inhibit tumor growth in vivo, results suggest that apigenin treatment significantly suppressed tumor growth and decreased the tumor volume and tumor weight. Our in vivo and in vitro study both indicate that apigenin can suppress cervical cancer development.

In the last decade, several studies have reported that apigenin could act as an estrogen receptor modulator, revealing the potential relation between apigenin and ER [26,27,28]. According to latest research, ERβ levels and/or the ERβ/ERα ratio decreases with ovarian carcinogenesis, indicating that loss of ERβ expression may be involved in carcinogenesis [29]. When it comes to ERα, accumulating evidence shows that the expression level of ERα is closely associated with estrogen-dependent growth and invasion in gynecologic cancer. For instance, as a direct target of the tumor suppressor microRNA (miR)-206, ERα usually causes down-regulation of minR-206 in ovarian cancer cell lines and tissues, whereas the introduction of miR-206 can inhibit cell proliferation and the invasion of cancer cells [30]. In this study, the histamine treatment decreased the ERβ/ERα ratio while apigenin showed the opposite influence. Specifically, Western blotting revealed that the expression level of ER showed the same changing trend of in vitro experiments, namely the ERβ/ERα ratio increased after the administration of apigenin. Similarly, cervical tumor development was inhibited in the nude mice injected with 2 mg/kg ER modulator (AZD9496 and DPN) and 1 mg/kg histamine. Our results suggest that apigenin can attenuate the abnormal ER signaling caused by histamine in vivo and in vitro.

According to a previous study, the hormonal treatment enables control of most climacteric symptoms, without any serious side effects [31]. As a kind of phytoestrogen, apigenin may have the potential to help the female body to retain safe levels of estrogen when histamine significantly decreases the estrogen in serum. Surprisingly, the secretion of 17β-estradiol (E2) in serum was found to be notably increased in the apigenin treated group. Apigenin had a similar chemical structure to E2, thus allowing it to competitively to bind to ER [32]. This competitive binding mechanism of E2 and apigenin may result in a reversal of the abnormal level of E2 in serum. Further studies should mainly focus on the comprehensive effects of apigenin on the human endocrine system.

The development of cancers is a complicated process involving various factors and pathways. Among the signaling pathways associated with apoptosis and autophagy, the PI3K/Akt/mTOR signaling pathway is well studied. Drugs inhibiting the PI3K/Akt/mTOR pathway are reported to have promoting effects on apoptosis in cancer cells [33]. Therefore, we mainly focused on the PI3K/Akt/mTOR signaling pathway in this study. The results showed that the expression levels of Akt, PI3K and mTOR in apigenin treated groups were much lower than the group treated with histamine alone. In the groups treated with 2 mg/kg ER modulator (AZD9496 and DPN) and 1 mg/kg histamine, Western blot results suggested that either inhibiting ERα or activating ERβ can inhibit the PI3K/Akt/mTOR signaling pathway. These findings indicated that the alternation of ER caused by apigenin induced cell apoptosis through PI3K/Akt/mTOR signaling pathway.

## 4. Materials and Methods 

### 4.1. Chemicals and Antibodies

Apigenin and histamine were from Sigma-Aldrich (St. Louis, MO, USA). The antibodies against Bax (1:1000), PI3K (1:1000), Akt (1:1000), and mTOR (1:1000) were obtained from Abbkine Scientific. (Wuhan, China). Antibodies for ERα (1:500), ERβ (1:500), p-PI3K (1:1000) and p-Akt (1:5000) were purchased from Abcam (Burlingame, city, CA, USA). AZD9496 (CAS No.: 1639042-08-2), PHTPP (CAS No.: 805239-56-9), PPT (CAS No.: 263717-53-9) and DPN (CAS No.: 1428-67-7) were obtained from MedChemExpress (Shanghai, China). FBS (Fetal bovine serum) was from Hyclone (Logan, UT, USA). Dulbecco’s modified Eagle medium (DMEM) was purchased from Gibco (Grand Island, NY, USA).

### 4.2. Cell Culture and Treatment

The human cervical cancer cell line (HeLa) was kindly provided from Cancer Hospital Chinese Academy of Medical Science (Beijing, China). The HeLa cells were routinely cultured in DMEM supplemented with 10% FBS and 1% (*v*/*v*) penicillin/streptomycin. Cells were maintained at 37 °C in a humidified incubator with 5% CO_2_. At 80% confluence, the HeLa cells were treated with different concentrations of histamine (1, 5, 10, 50, 100 ng/mL) and apigenin (0.1, 0.5, 1, 5, 10 μM) for 48 h. Apart from this, AZD9496, PHTPP, PPT and DPN at the concentration of 1 μM were added to other HeLa cells and incubated for 48 h.

### 4.3. Determination of Cell Proliferation

Cell proliferation was measured using Cell Counting Kit-8 (Beyotime, Beijing, China) according to the manufacturer’s instructions. Briefly, 2 × 10^4^ cells were seeded into 96-well plates. After treatment, each well had 90 μL of medium and 10 μL of CCK-8 solution added, and cells were then incubated at 37 °C for 1 h. The absorbance was measured at 570 nm using a Microplate Reader. The value of absorbance was used to calculate the cell proliferation.

### 4.4. Animal Model

The female BALB/c nude mice (6-weeks-old) in our research were purchased from Vital River Laboratories, Inc. (Beijing, China) and housed in the specific pathogen-free (SPF) animal laboratory of College of Food Science and Nutritional Engineering, China Agricultural University (Beijing, China). The animal laboratory was maintained at a temperature of 22 ± 1 °C, humidity of 55 ± 5%, a 12 h light/dark cycle and air exchanges at 15 times/h. Feed and water were supplied ad libitum. All animal experiments in this study were approved by the Animal Experimental Welfare and Ethical Inspection Committee in China Agricultural University.

HeLa cells (2 × 10^6^) were re-suspended in 100 μL PBS and then injected subcutaneously into the lower right-side flanks of mice to establish tumors. After 6 days of initial implantation, sixty female BALB/c nude mice (6-weeks-old) were randomly divided into six groups with equal body weight (n = 10/group). Groups and treatment are listed in Table 1. The dose and administration approach were selected according to previous studies [24]. Tumor volumes were measured with calipers every 3 days and tumors volumes (V) were calculated using the formula: V = 1/2 length × (width)^2^. All mice were sacrificed at 30 days and the tumors were dissected and weighed.

### 4.5. ELISA Assays for Serum Cytokine Levels and Serum Estradiol Level

Serum samples which collected from orbital sinus were used to quantified VEGF, TNF-α and estradiol using commercial mouse ELISA kit (Abcam, Cambridge, MA, USA).

### 4.6. Immunohistochemistry Analysis

Normal tissues and tumor tissues were embedded in paraffin and sectioned at a thickness of 4 μm. Sections of tumor were incubated with respective antibodies at 4 °C overnight after hydration and antigen retrieval. Then, biotin-conjugated secondary anti-mouse IgG was used to incubated with washed tumor sections for 30 min at room temperature. Diaminobenzidine was used to visualize sections according to the manufacturer’s instructions. Results were calculated as follows:

IHS = A × B(1)

where A is the score for percentage of positive cells (0~1%—0, 1~10%—1, 10~50%—2, 50~80%—3, 80~100%—4) and B is positive cell color intensity grade (negative—0, weak positive—1, positive—2, strong positive—3).

### 4.7. Real-Time PCR

After extraction by Trizol reagent (TaKaRa), the total RNA in the HeLa cells was reverse transcribed using a cDNA reverse transcription kit according to the manufacturer’s instructions. Primer sequences of selected genes are shown in Table 2. β-actin was used as an internal control.

### 4.8. Western Blot Analysis

Briefly, HeLa cells were homogenized in protein lysate buffer and centrifuged at 12,000× *g* for 15 min at 4 °C. The protein concentrations were quantified by using the Bradford protein assay kit (Beyotime, Beijing, China). After that, protein samples were separated by SDS-PAGE, transferred to polyvinylidene fluoride membranes and blocked with fresh 5% nonfat milk for 2 h at room temperature. The membranes were then incubated with specific primary antibody at 4 °C overnight. Horseradish peroxidase-conjugated secondary antibodies were used to incubated with membranes which were washed with TBST. Proteins were visualized by using chemiluminescence reagents and the signal was detected by a gel documentation system (GelDoc-It 310 Imaging System).

### 4.9. Statistical Analysis

All data are presented as the mean values ± SEM from three independent biological replicates. Statistical significance was determined by one-way analysis of variance (ANOVA) or student’s *t* test using GraphPad Prism 8.01 (GraphPad Software, Inc., San Diego, CA, USA). A value of *p* < 0.05 was considered to indicate a statistically significant different.

## 5. Conclusions

In conclusion, we demonstrated that histamine induced HeLa cell proliferation in vivo and in vitro, while apigenin showed the opposite effects. In addition, apigenin was found to attenuate the abnormal ER signaling caused by histamine, which resulted in the inhibition of the PI3K/Akt/mTOR pathway. Moreover, this study is the first to show that apigenin can inhibit histamine-induced cervical cancer tumor growth by regulating ER expression. This result may provide a new insight into the anti-tumor activity of apigenin and a series of experimental and theoretical bases which can help us to better utilize phytoestrogens in cancer therapy.

## Figures and Tables

**Table 1:** Treatment groups and doses.

Groups	Descriptions	Comment
Control	No HeLa cells injected	Negative control to see the effects of other treatment
HeLa	Injected with 2 × 10^6^ HeLa cells	Positive control to mimic cervical cancer
HA	Intraperitoneally injected with 1 mg/kg histamine every 3 days	Test group to study the effects of histamine on cervical cancer
HA + APG	Intraperitoneally injected with 1 mg/kg histamine and 100 mg/kg apigenin every 3 days	Test group to study the effects of apigenin on cervical cancer
HA + AZD9496	Intraperitoneally injected with 1 mg/kg histamine and 2 mg/kg AZD9496 every 3 days	Test group to study the effects of ERα on cervical cancer
HA + DPN	Intraperitoneally injected with 1 mg/kg histamine and 2 mg/kg DPN every 3 days	Test group to study the effects of ERβ on cervical cancer

**Table 2:** Primers used for RT-PCR.

Gene	Primer Sequence (5′–3′)	Size of Product
β-actin	CTCGCCTTTGCCGATCC	258 bp
	GGGGTACTTCAGGGTGAGGA	
ERα	ATGCGCTGCGTCGCCTCTAAC	78 bp
	CGCAGGGCAGAAGGCTCAGA	
ERβ	AGCGCGGAGGCTGCGAGAAAT	56 bp
	CCTGCTCTTCGCCCTGCAAGTT	
